# Effects of extreme meteorological conditions in 2018 on European methane emissions estimated using atmospheric inversions

**DOI:** 10.1098/rsta.2020.0443

**Published:** 2022-01-24

**Authors:** R. L. Thompson, C. D. Groot Zwaaftink, D. Brunner, A. Tsuruta, T. Aalto, M. Raivonen, M. Crippa, E. Solazzo, D. Guizzardi, P. Regnier, M. Maisonnier

**Affiliations:** ^1^ NILU – Norsk Institutt for Luftforskning, Kjeller, Norway; ^2^ Empa, Swiss Federal Laboratories for Materials Science and Technology, Dübendorf, Switzerland; ^3^ Climate System Research, Finnish Meteorological Institute, Helsinki, Finland; ^4^ Institute for Atmospheric and Earth System Research/Physics, Faculty of Science, University of Helsinki, Helsinki, Finland; ^5^ European Commission Joint Research Centre, Ispra, Italy; ^6^ Biogeochemistry and Modeling of the Earth System (BGEOSYS), Université Libre de Bruxelles, Brussels, Belgium

**Keywords:** methane, emissions, Europe, atmospheric inversion, CH_4_, anomaly

## Abstract

The effect of the 2018 extreme meteorological conditions in Europe on methane (CH_4_) emissions is examined using estimates from four atmospheric inversions calculated for the period 2005–2018. For most of Europe, we find no anomaly in 2018 compared to the 2005–2018 mean. However, we find a positive anomaly for the Netherlands in April, which coincided with positive temperature and soil moisture anomalies suggesting an increase in biogenic sources. We also find a negative anomaly for the Netherlands for September–October, which coincided with a negative anomaly in soil moisture, suggesting a decrease in soil sources. In addition, we find a positive anomaly for Serbia in spring, summer and autumn, which coincided with increases in temperature and soil moisture, again suggestive of changes in biogenic sources, and the annual emission for 2018 was 33 ± 38% higher than the 2005–2017 mean. These results indicate that CH_4_ emissions from areas where the natural source is thought to be relatively small can still vary due to meteorological conditions. At the European scale though, the degree of variability over 2005–2018 was small, and there was negligible impact on the annual CH_4_ emissions in 2018 despite the extreme meteorological conditions.

This article is part of a discussion meeting issue ‘Rising methane: is warming feeding warming? (part 2)’.

## 1. Introduction

Methane (CH_4_) is the second most important well-mixed greenhouse gas (WMGHG) after carbon dioxide (CO_2_) and is responsible for 17% of the direct radiative forcing by all WMGHGs [1]. Atmospheric CH_4_ has increased since 2007, after a period of relative stability in the late 1990s and early 2000s. From 2014, CH_4_ increased very rapidly at rates not seen since the 1980s [2]. This is a particular concern since CH_4_ is a potent WMGHG with a global warming potential 28 times that of CO_2_ on a 100-year time scale [1], and if current trends continue, CH_4_ poses a serious threat to the aim of the Paris agreement to limit global warming to less than 2°C [3]. The cause of the recent increase in CH_4_ is not well understood, and various explanations have been proposed including a decrease in the primary atmospheric sink due to the hydroxyl radical [4], an increase in biogenic emissions such as from agriculture and wetlands [5,6] and a combined increase in biogenic and fossil fuel emissions with a concurrent decrease in biomass burning emissions [7,8]. Recent studies, however, indicate that a decrease in the hydroxyl radical is unlikely [9–11], meaning that the CH_4_ increase is more likely due to significant changes in the sources rather than the sinks.

Changes in both anthropogenic and natural emissions may have contributed to the observed CH_4_ increase. In particular, natural biogenic sources of CH_4_, such as wetlands and peatlands, are sensitive to changes in rainfall and temperature and are potential positive feedback mechanisms in the climate system [12–14]. However, the extent to which these natural sources have contributed to the recently observed CH_4_ trend is unclear [15]. While anthropogenic biogenic sources, like agriculture and waste, are very much reliant on human activity, they are also sensitive to changes in environmental conditions. In particular, CH_4_ emissions from rice cultivation and manure are strongly dependent on temperature with a Q10 factor (i.e. the factor by which emissions increase with a 10° increase in temperature) similar to that of wetland emissions [16]. Emissions from landfills, on the other hand, have a more complex relation to temperature being positively correlated with temperature when soils are moist but negatively correlated under dry conditions [17]. The possible net response of anthropogenic CH_4_ emissions to temperature and soil moisture changes at national or regional scales, however, is unknown.

In national emissions reporting, following the Intergovernmental Panel on Climate Change (IPCC) guidelines, only anthropogenic sources are considered, and the reported emissions do not account for possible temporal variations due to meteorological conditions, at least for Tier 1 methods which are predominantly used [18]. This is due to the fact that the emissions are estimated based on atemporal emission factors multiplied with annual activity data. Thus, in comparing national reported emissions with estimates based on atmospheric observations (a ‘top-down’ approach), there may be significant differences due to seasonal and inter-annual variations in the anthropogenic sources, which are not accounted for in the emission inventories. This represents one advantage of top-down approaches to emissions estimates, namely, they include all variations in the emissions, those due to changes in human activity as well as those due to environmental factors.

**Figure 1.**
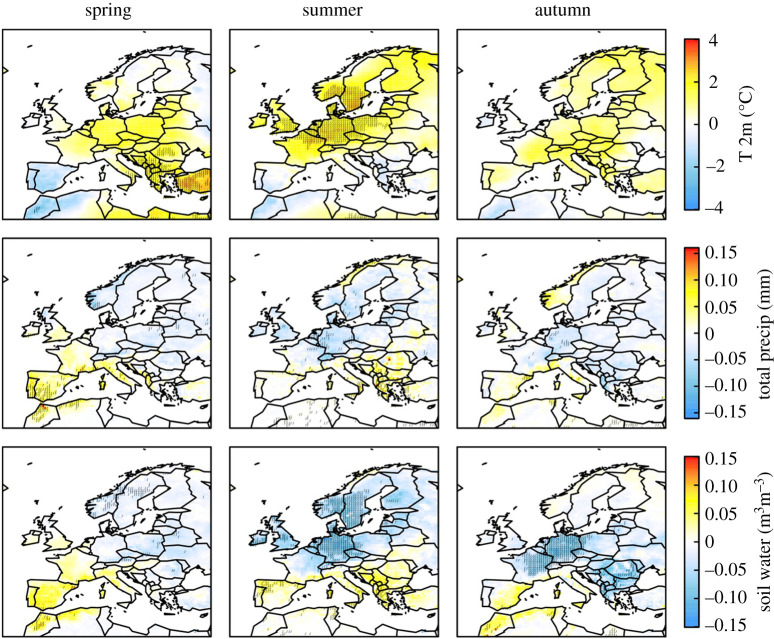
Anomalies in air temperature at 2 m (°C), total precipitation (mm) and SWV for 0–7 cm depth (m^3^m^−3^) for 2018 compared to 2005–2018 from ECMWF ERA5. The stippling indicates grid cells where the anomaly is more than two standard deviations from the mean. (Online version in colour.)

In this study, we use the top-down approach, specifically atmospheric inversions, to estimate CH_4_ emissions from 2005 to 2018 for geographical Europe with the aim of determining the possible variability in anthropogenic and natural sources due to meteorological conditions. We include estimates from four different atmospheric inversion frameworks used in the VERIFY project (http://verify.lsce.ipsl.fr) to assess the systematic uncertainty in the emissions from this approach. In particular, we examine CH_4_ emissions in 2018 as Europe experienced extreme meteorological conditions in this year with a heatwave and drought affecting regions north of the Alps while regions south of the Alps experienced a wetter than usual summer [19,20]. Figure 1 shows the anomalies in temperature, precipitation and soil water for spring (March–May), summer (June–August) and autumn (September–November) of 2018 with respect to mean values for 2005–2018 using reanalysis data from the European Centre for Medium-Range Weather Forecasts (ECMWF). In this paper, we first present the inversion methodology and the input data used, and then an overall assessment of the inversion results. Subsequently, we discuss the CH_4_ emission anomalies in 2018 compared to 2005 to 2017 and their possible causes.

## 2. Methodology

### (a) Overview of atmospheric inversions

In this study, we use four atmospheric inversion frameworks, which are all based on Bayesian statistics to find the optimal CH_4_ fluxes, that is, those which have the maximum posterior probability given the observations and prior information. All frameworks used in this study assume that the observation and prior fluxes are normally distributed, thus the problem of finding the optimal fluxes is equivalent to finding the fluxes that minimize the following cost function [21]:

2.1 $$J(x)={\left(x-x_{b}\right)}^{T}{\text{B}}^{-1}(x-x_{b})+{\left(H(x)-y\right)}^{T}{\text{R}}^{-1}(H(x)-y)$$

where *x* and *x_b_* are vectors of the optimal (posterior) and prior fluxes, respectively, *y* is a vector of the observed atmospheric mole fractions of CH_4_, and *H*(*x*) is the atmospheric transport. The matrices, B and R, describe the error covariance of the prior fluxes and observations, respectively. For the observations, R is a diagonal matrix, that is, it is assumed that the observations assimilated into the inversion are not correlated. The four inversion frameworks include two different atmospheric transport models (FLEXPART [22] and TM5 [23]), two regional inversions (FLEXINVERT [24] and FLEXPART-ExKF [25]) and two global inversions (TM5-4DVAR [26] and CarbonTracker Europe CH_4_ [27]) with Europe covered at higher resolution (table 1). Details about the individual inversion frameworks are provided in the electronic supplementary material, information.

**Table 1.** Overview of the inversion frameworks. The resolution over Europe is for the CH_4_ fluxes output from the inversions.

	FLEXINVERT	FLEXKF	TM5-4DVAR	CTE
full name	FLEXINVERT	FLEXPART-ExKF	TM5-4DVar	CarbonTracker Europe CH4
resolution Europe	0.25° × 0.25°^a^	0.5° × 0.5°	1° × 1°	1° × 1°
temporal resolution	monthly	monthly	monthly	monthly
transport model	FLEXPART-v10.3	FLEXPART-v9.1	TM5	TM5
model domain	Europe	Europe	global	global
European domain^b^	15°E to 45°W, 33°N to 83°N	15°E to 35°W, 33°N to 73°N	18°E to 42°W, 32°N to 64°N	EU27 + UK countries
algorithm	analytical	ExKF^c^	4D-Var	EnKF^d^
observation dataset	core	core	core	core
background	Flexpart-CTM	TM5-4DVAR	N/A	N/A
reference	Thompson *et al*. [24]	Brunner *et al*. [25]	Bergamaschi *et al*. [26]	Tsuruta *et al*. [27]

^a^A variable resolution was used from 0.25° to 2°.

^b^For the global models this is the domain covered at 1° × 1° resolution.

^c^ExKF is the Extended Kalman filter.

^d^EnKF is the Ensemble Kalman filter.

### (b) Atmospheric observations

**Figure 2.**
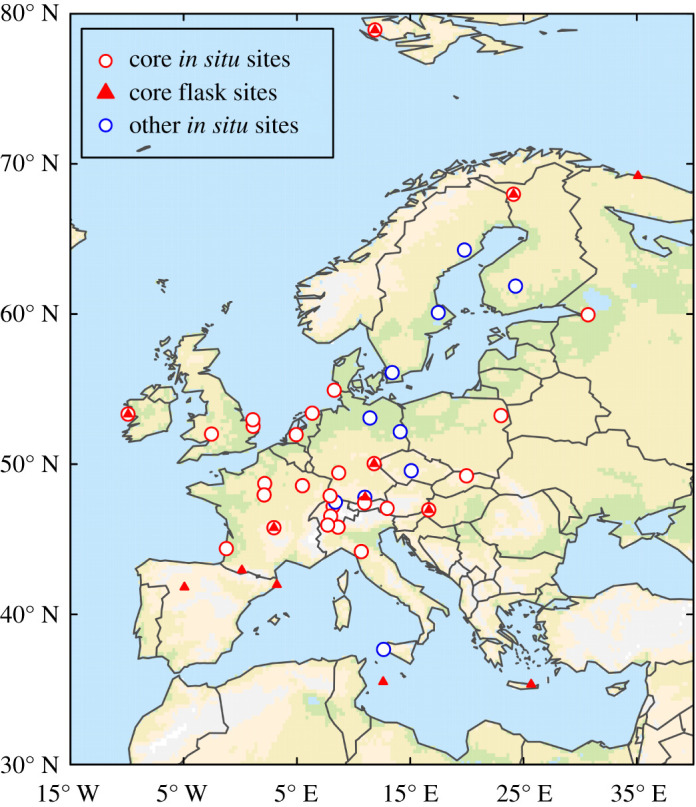
Map of atmospheric observation sites included in the Core (red) and Inclusive (red and blue) datasets with *in situ* (open circles) and flask (triangles) measurements (note some *in situ* and flask sites are co-located).

Atmospheric observations of CH_4_ mole fractions were compiled from the following sources: (i) the InGOS project harmonized dataset, which approximately covers the period from 2005 to 2014; (ii) the ICOS-Atmosphere network; (iii) the World Data Centre for Greenhouse Gases (WDCGG); (iv) the NOAA ESRL discrete sampling network; (v) the EBAS database and (vi) personal communications from station principle investigators. Information on where to obtain these data is given in the Data Availability section. Based on these, a dataset of 61 records from 54 locations for 2005–2018 was compiled, which included 16 discrete flask sampling sites and 45 *in situ* sampling sites (figure 2; electronic supplementary material, table S1). The measurements are all reported as dry air mole fractions and were calibrated to the WMO CH4X2004(A) scale, except the *in situ* measurements at the site Mace Head (MHD), which used the Tohoku University scale. The conversion factor from this scale to WMO CH4X2004 is 1.0003, thus the difference between these scales is very small (e.g. at 1900 ppb this error is 0.6 ppb) and smaller than other sources of error inversion estimates.

Since the observation network changes over time (some sites were discontinued while others started after 2005), a subset of 31 timeseries (at 26 locations) covering a minimum of 9 years between 2005 and 2018 was chosen and is from here on referred to as the ‘core’ dataset (electronic supplementary material, table S1). The motivation for using the core dataset is that changes in the observation network over time mean that the degree of independence from the prior estimates will also change over time and may introduce some artificial year-to-year variability in the posterior fluxes. The threshold of 9 years was a compromise between having long records versus the overall number of sites included in the core dataset. All inversion results presented are from using the core dataset. One inversion framework, CTE, was run using both the core dataset and the dataset including the additional sites, and the comparison of these is shown in the electronic supplementary material, figure S1.

Measurement uncertainties were provided with some of the data and represent the repeatability of the measurement based on analyses of a gas standard. This information, where provided, was considered in estimating the full observation space uncertainty, and where it was not provided a measurement uncertainty of 2–3 ppb was assumed in line with the World Meteorological Observation Global Atmospheric Watch (WMO GAW) target precision for CH_4_. The observation space uncertainty is generally much larger than the measurement uncertainty, due to model representation errors, and was estimated separately by the individual inversion frameworks for each observation, but ranged from about 5 to 100 ppb (see electronic supplementary material, information). In all inversion frameworks, only daytime observations were assimilated for low-altitude sites and nighttime observations for mountain sites. In three of the inversion frameworks, the averaged daytime/nighttime observations were used, while in TM5-4DVAR, hourly observations for daytime/nighttime were used.

### (c) Flux estimates and boundary conditions

All inversions used the same prior flux estimates, which were prepared at 0.5° × 0.5° and monthly resolution from a number of models (electronic supplementary material, figure S2 and table S2). The prior fluxes included estimates for the following:

— Anthropogenic emissions: including agriculture, waste, fossil fuels (incomplete combustion and fugitive emissions), biofuel and biomass burning. These were provided by the Emission Database for Global Atmospheric Research (https://edgar.jrc.ec.europa.eu/overview.php?v=verify_h2020, EDGAR-v6) at monthly and 0.1° × 0.1° resolution and covered the period 2005–2018.
— Natural emissions from peatlands, inundated and mineral soils, as well as uptake via oxidation in mineral soils. These were provided by the land surface model, JSBACH-HIMMELI, run using merged Climate Research Unit and Japanese Reanalysis (CRU-JRA) climate data (as used in the Global Carbon Project (GCP) CH_4_ budget) and with daily and 1.875° × 1.875° resolution [28,29,30].
— Natural emissions from lakes were provided by an empirical model using the HydroLakes database and were an annual climatology with 0.1° × 0.1° resolution [29,30].
— Natural emissions from geological manifestations and onshore seepage were based on Etiope *et al*. [31] and were an annual climatology with 1.0° × 1.0° resolution prepared for the GCP CH_4_ Budget 2020 [32].
— Natural emissions from termites were provided by the GCP CH_4_ Budget 2016 and were provided as an annual climatology and at 1.0° × 1.0° resolution [33].
— Ocean fluxes were taken from Weber *et al*. [34] and were provided as a monthly climatology at 0.25° × 0.25° (available from: https://figshare.com/articles/dataset/ocean_ch4_nc/9034451).

In our analyses (presented in §4), we also refer to flux estimates from the Global Dynamic Vegetation Model, LPX-Bern [35,36]. LPX-Bern was driven using CRU climate data and run in a prognostic mode in which the inundated and peatland areas were determined dynamically from the climate input. We chose to use JSBACH-HIMMELI (in the prior) and LPX-Bern (in the analyses), as these models both provide CH_4_ fluxes not only for wetlands but also for mineral soils, and they both have a specific treatment of peatlands (a subclass of wetlands) using peatland-specific carbon dynamics [29,30,35].

For the regional inversions (FLEXINVERT and FLEXKF), a boundary condition is needed to account for the influence of transport of CH_4_ from outside the regional domain (and from outside the time interval of the Lagrangian model calculations) to the observation locations and times, this is from here on referred to as the ‘background’. In FLEXKF, the background was calculated using Rödenbeck *et al*.'s [37] two-step method using the optimized fields of CH_4_ mole fractions from TM5-4DVAR (see electronic supplementary material, information for further details). In FLEXINVERT, it was calculated by coupling the endpoints of the Lagrangian particle trajectories (calculated globally) to optimized CH_4_ fields from the global model, FLEXPART-CTM according to the method of Groot Zwaaftink *et al*. [38] and transporting the influence of fluxes outside the regional domain to the observation locations and times (see electronic supplementary material, information for further details).

## 3. Results

### (a) Assessment of the inversion results

**Figure 3.**
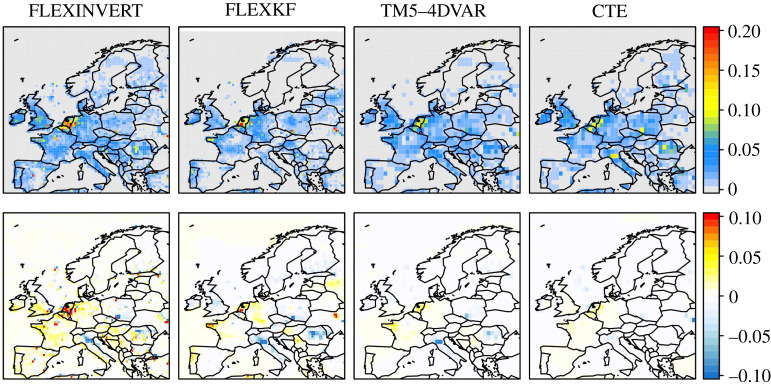
Annual mean CH_4_ fluxes (top) and posterior-prior flux increments (bottom) for the example year 2018. The fluxes are all plotted at 0.5° × 0.5° and in units of g m^−2^ day^−1^. (Online version in colour.)

Figure 3 shows maps of the annual mean CH_4_ fluxes, as well as the difference with respect to the prior estimate, from all four inversion frameworks for the example year 2018. The two regional inversions, FLEXINVERT and FLEXKF, exhibit much finer spatial structure as expected since these inversions were run at a finer resolution (specifically for FLEXINVERT on a grid of variable resolution (from 0.25° × 0.25° to 2.0° × 2.0°) and for FLEXKF on a grid of 0.5° × 0.5° resolution) while the global inversions, TM5-4DVAR and CTE, were run at 1.0° × 1.0° over Europe (and coarser outside of Europe). Despite the differences in resolution, the emission distribution between inversions is very similar with large emissions in the Netherlands and Belgium, and low emissions in Northern Europe. The inversions also show very similar patterns of flux differences (or increments) with increases with respect to the prior estimates over the Netherlands and western France and decreases over Italy, Poland and, for three of the inversions, Romania. The decrease over Poland coincides with the Silesian coal-mining region and may indicate that coal-mining emissions are over estimated in EDGAR-v6.0 (electronic supplementary material, figure S3). Whereas the decreases over Italy and Romania coincide with where the prior estimate has high geological emissions and thus may indicate that the geological emissions are overestimated in these areas (electronic supplementary material, figure S3). The inversions differ, however, in how far the posterior fluxes depart from the prior estimates. The global models have smaller flux increments compared to the two regional ones, for example, the mean increments over the Netherlands are 0.14 Tg y^−1^ and −0.09 Tg y^−1^ for TM5-4DVAR and CTE, respectively, compared with 0.78 Tg y^−1^ and 0.52 Tg y^−1^ for FLEXINVERT and FLEXKF. In the case of CTE, the smaller increments correspond to a smaller *a posteriori* improvement in the agreement to the observed CH_4_ mole fractions compared to the other inversions, and a smaller uncertainty reduction, which indicates that this inversion had fewer effective degrees of freedom (electronic supplementary material, figures S4 and S5).

Three of the inversion frameworks calculated the posterior flux uncertainty, from which the uncertainty reduction can be derived as: 1 – *σ*_post_/*σ*_prior_, where *σ*_post_ and *σ*_prior_ are the posterior and prior uncertainties, respectively. The uncertainty reduction in all three inversions is high in Western Europe, reaching up to approximately 50%, and is generally lower in Southern Europe, which is a direct result of the distribution of the atmospheric observations (electronic supplementary material, figure S5).

**Figure 4.**
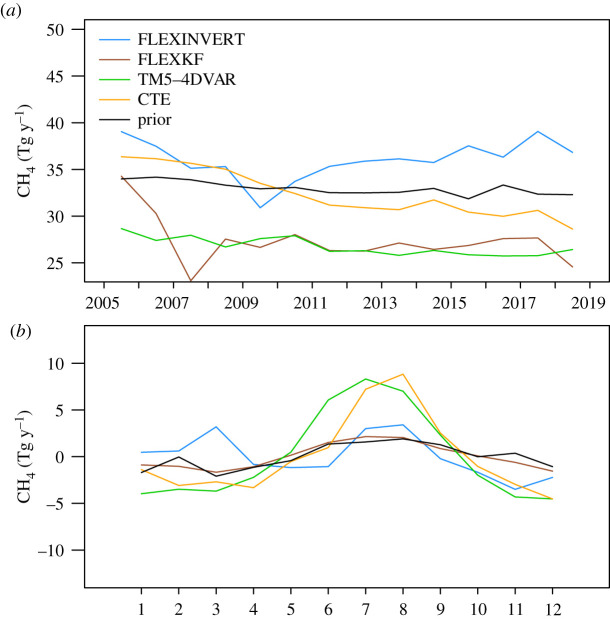
Annual mean (*a*) and seasonal anomaly (*b*) of the area integrated CH_4_ source for Europe (EU27 + UK) in units of Tg y^−1^. The seasonal anomaly was calculated for each inversion by first subtracting the multi-annual trend from the data and second by averaging each month over the period 2005–2018.

For a more quantitative comparison of the inversion results, we calculated the annual integrated emission for the region EU27 + UK (figure 4; electronic supplementary material, table S3). We chose the EU27 + UK as emissions for this region are reported in other studies allowing the comparison of our results also to previous estimates. FLEXKF and TM5-4DVAR find somewhat lower total emissions for 2005 to 2018 with 27.3 and 26.8 Tg y^−1^, respectively, compared with the prior estimate of 33.0 Tg y^−1^, whereas FLEXINVERT and CTE remain on average closer to the prior with 36.0 and 32. Tg y^−1^, respectively. The similarity of FLEXKF and TM5-4DVAR may in part be due to the fact that FLEXKF used output from TM5-4DVAR in the description of the background, which is discussed below. Another important difference between the inversions is that the global inversions (TM5-4DVar and CTE) find a significant negative trend in the emissions from 2005 to 2018 of 0.19 and 0.57 Tg y^−1^, respectively, whereas the regional ones do not. The range of mean emission estimates for the EU27 + UK across the inversions is considerable, from 26.8 to 36.0 Tg y^−1^ or equivalently −11 to +17% of the mean value. However, the inversions result in considerable reductions in the emission uncertainty for EU27 + UK with respect to the prior, ranging from 37% to 85%, for the three inversions that calculate this. The lower end of the emission range is close to the mean of 26.8 Tg y^−1^ for the seven inversions compared in Bergamaschi *et al*. [39] for 2006–2012 and to the regional inversions compared in Petrescu *et al*. [40] with a mean of 28.8 Tg y^−1^ for 2011–2015, although these studies are not entirely independent as they include some of the same inversion frameworks as this study.

A small seasonal cycle is present in the prior flux estimate with a broad maximum in summer and amplitude of 2.5 Tg y^−1^ (calculated as the mean difference between December–February and June–August) (figure 4). The global inversions TM5-4DVAR and CTE find considerably larger seasonal amplitudes of 11.1 and 8.7 Tg y^−1^, while FLEXINVERT and FLEXKF find amplitudes close to that of the prior estimate with 2.2 and 2.5 Tg y^−1^, respectively. Noteworthy, however, is that when using the prior fluxes the global model, TM5 (used in TM5-4DVAR and CTE) overestimates the amplitude of the atmospheric seasonal cycle in CH_4_ with a lower summer minimum compared to the observations at a number of sites (i.e. Hohenpeissenberg (HPB), Ispra (IPR), Heidelberg (HEI) and Hegyhatsal (HUN)), whereas the regional model, FLEXPART, does not (electronic supplementary material, figure S6). Since the summer minimum is due to the combined processes of atmospheric loss by OH oxidation and a deepening of the mixed layer and of the troposphere, this could indicate a bias in the atmospheric transport or chemistry of the global model. In which case, this would explain why the global inversions (using TM5) find a larger summer maximum and seasonal amplitude, since larger summer emissions are needed to match the observed atmospheric seasonal cycle.

The background calculation in the regional inversions (FLEXINVERT and FLEXKF) is another source of uncertainty and may at least partly explain why the results of these inversions differ in their estimates for the EU27 + UK region. Comparisons of the background and observed CH_4_ mole fractions for observation times when the contribution from fluxes within the European domain is close to zero (defined as when the absolute difference between the prior modelled and background mole fraction is less than 5 ppb) suggest that the background in FLEXINVERT is underestimated by an average of −18 ppb (standard deviation of 15 ppb) compared to −7 ppb (standard deviation of 15 ppb) in FLEXKF, which could mean FLEXINVERT has a positive bias for the flux estimate over Europe (electronic supplementary material, figure S7). The fact that FLEXKF used TM5-4DVAR output to determine the background means that both FLEXKF and TM5-4DVAR have the same budget of CH_4_ entering into the European domain via atmospheric transport.

### (b) Anomalies in 2018

**Figure 5.**
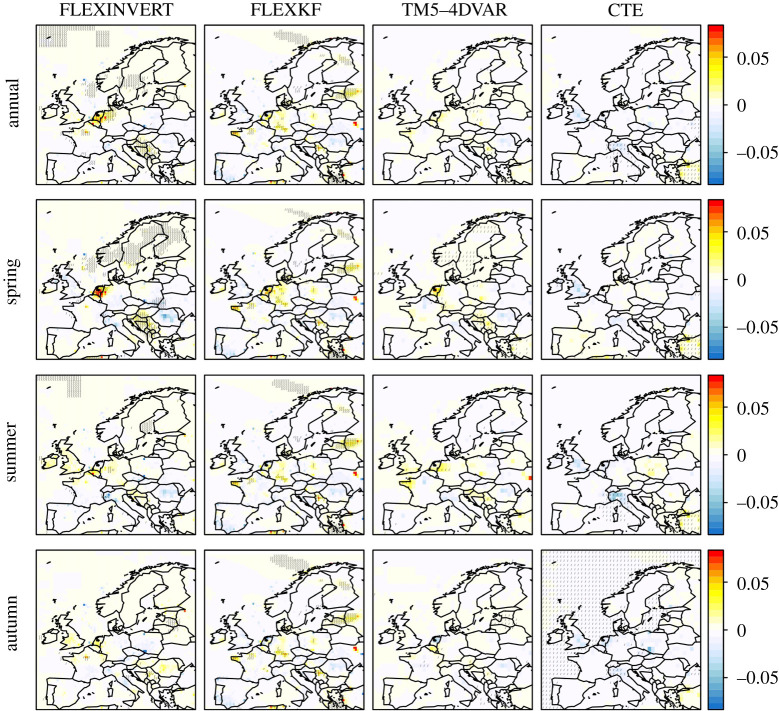
Annual, spring (March–May), summer (June–August) and autumn (September–November) anomalies in CH_4_ fluxes for 2018 compared to 2005–2018 in units of g m^−2 ^day^−1^. The stippling indicates grid cells where the anomaly is more than two standard deviations from the mean. (Online version in colour.)

The annual and seasonal CH_4_ flux anomalies in 2018 with respect to the mean for 2005–2017 are shown in figure 5. A positive anomaly in the annual mean is seen in three of the inversions (but not in CTE) over the Netherlands and northwest Germany, but is only significant (i.e. outside two standard deviations of the mean) in FLEXINVERT. A positive annual anomaly is also seen over Serbia. The anomalies are stronger when considering only the spring (March–May). Since the Netherlands and Serbia are the only regions to have consistent anomalies across at least three of the inversions, we focus on the results for these two regions. The regionally integrated fluxes and flux anomalies for the Netherlands and Serbia for 2005–2018 are also shown in the electronic supplementary material, figures S8 and S9 and table S3.

**Figure 6.**
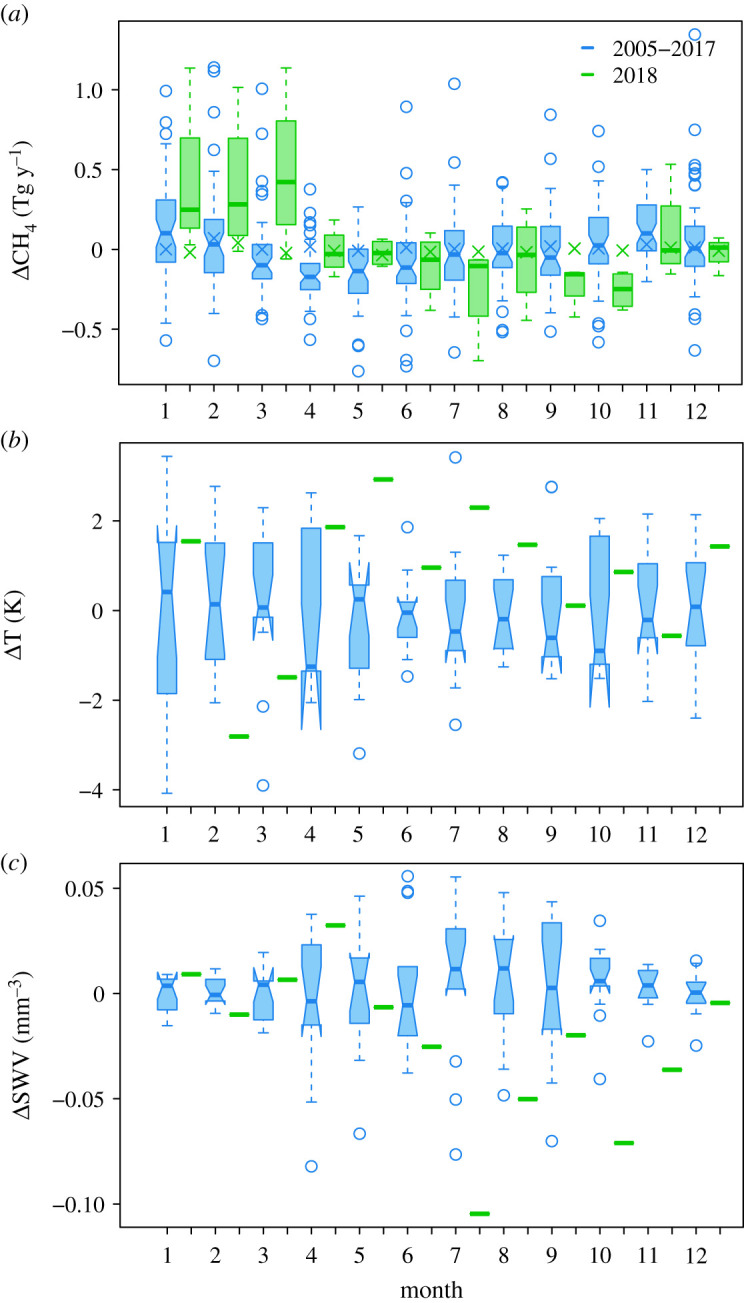
Median and interquartile ranges of the monthly CH_4_ anomaly in the Netherlands (*a*), for temperature anomalies (*b*) and for SWV anomalies. (*c*) Shown are data for 2005–2017 (blue) and for 2018 (green). Also shown is the median prior CH_4_ source for 2005–2017 and the prior value for 2018 (crosses).

Figure 6 shows median and interquartile ranges of the monthly CH_4_ source for the Netherlands from all inversions (and the prior estimates) for the years 2005 to 2017 versus for the year 2018. Also shown are the median and interquartile ranges of the monthly air temperature at 2 m and soil water volume (SWV) at 0–7 cm depth from the ECMWF ERA5 reanalysis. The prior source estimates for 2005 to 2017 versus 2018 are not significantly different. To test the significance of the difference in the monthly CH_4_ source from the inversions, we use a Wilcoxon rank-sum test (in R version 3.2.3) with the null hypothesis that the CH_4_ sources for the two samples, i.e. for 2005–2017 versus 2018, are the same. In this test, we give equal weight to all inversion results as we do not have any strong basis not to do so. Weighting the emissions by the posterior uncertainty is also unsatisfactory as this depends strongly on the prior and observation uncertainties, which is different in each inversion. (The posterior compared to the prior uncertainty is a good indicator for the observational constraint in an inversion but not a good indicator for the overall validity of the posterior emissions). We find that the CH_4_ source was with 95% confidence higher for March, and with 90% confidence higher for April, 2018 than for the previous 13 years by 0.53 ± 0.35 and 0.15 ± 0.25 Tg y^−1^, equivalently 65 ± 43% and 19 ± 32% of the prior annual source. By contrast, for September and October 2018, the CH_4_ source was lower with 90% confidence by 0.22 ± 0.07 Tg y^−1^ and lower with 95% confidence by 0.30 ± 0.27 Tg y^−1^, respectively, or 27 ± 9% and 37 ± 33% of the prior annual source. However, there was no difference in the annual mean CH_4_ source for 2018 versus that for 2005–2017.

**Figure 7.**
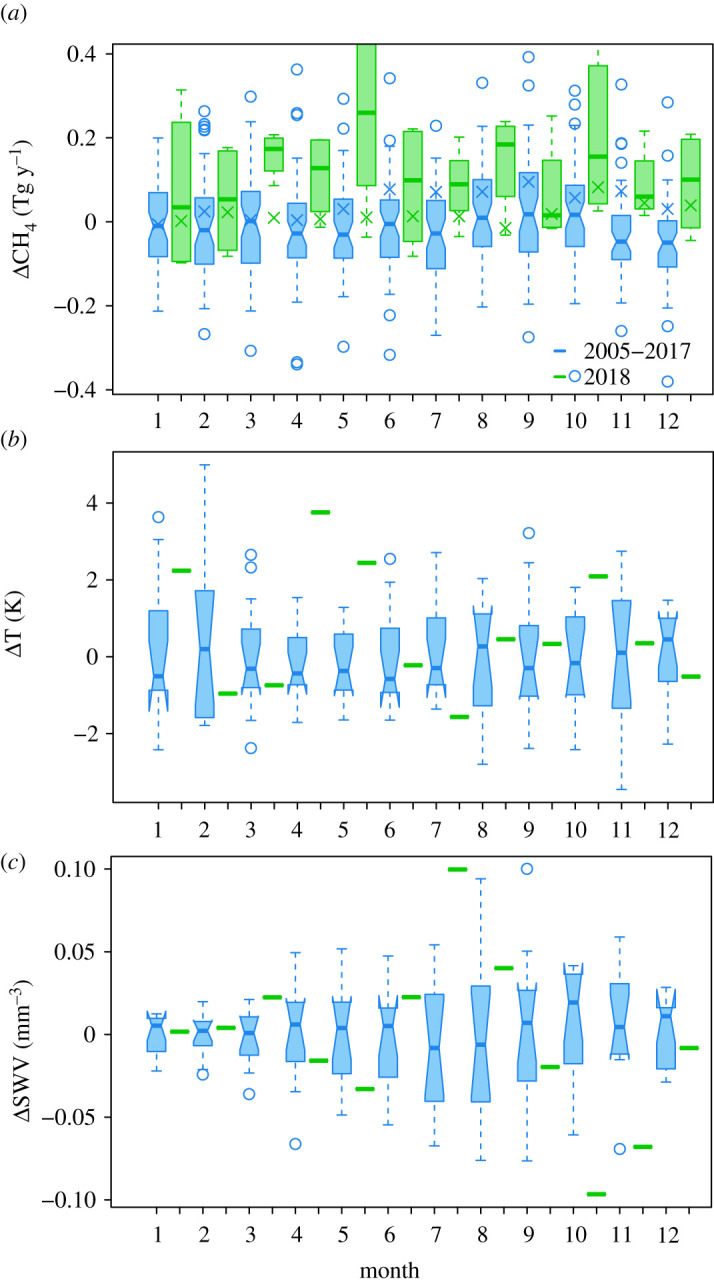
Median and interquartile ranges of the monthly CH_4_ anomaly in Serbia (*a*), for temperature anomalies (*b*) and for SWV anomalies. (*c*) Shown are data for 2005–2017 (blue) and for 2018 (green). Also shown is the median prior CH_4_ source for 2005–2017 and the prior value for 2018 (crosses).

Similar to figure 6, we show the median and interquartile ranges of the monthly CH_4_ source for Serbia in figure 7. For Serbia, the CH_4_ source was with 95% confidence higher for March–May, July and October–December by an average of 0.20 ± 0.18, 0.12 ± 0.07 and 0.15 ± 0.14 Tg y^−1^, respectively, or equivalently, 44 ± 42%, 27 ± 16% and 33 ± 36% of the prior annual source. Moreover, the annual mean source for Serbia in 2018 was 0.13 ± 0.15 Tg y^−1^ (33 ± 38%) higher than the prior mean of the previous 13 years.

## 4. Discussion

In 2018, Europe north of the alps experienced a warm spring, followed by an exceptionally hot and dry summer with soil water deficits persisting into the autumn [19]. The epicentre of the summer heatwave and drought was Western Europe and southern Scandinavia, and, in particular, Germany and the Benelux region (figure 1).

**Figure 8.**
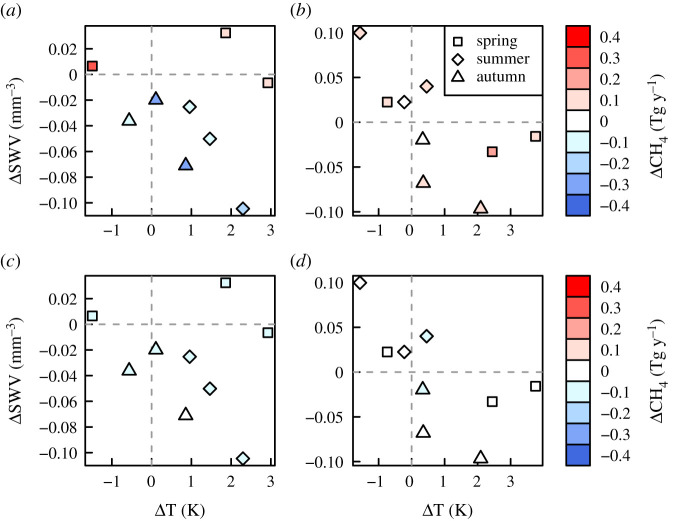
Distribution of the 2018 monthly mean CH_4_ anomalies (mean of all inversions) by temperature and SWV for (*a*) the Netherlands and (*b*) Serbia. Distribution of the 2018 monthly CH^4^ anomalies from the prior fluxes for (*c*) the Netherlands and (*d*) Serbia. The months are grouped into spring (March–May, squares), summer (June–August, diamonds) and autumn (September–November, triangles). Note the winter months are not shown since emissions in winter are generally not driven by microbial processes and hence are not affected by temperature or soil moisture anomalies. (Online version in colour.)

For the Netherlands, the positive CH_4_ anomaly in April corresponded to positive anomalies in temperature and soil moisture (figure 8), and it is possible this combination provided optimal conditions for microbial emissions of CH_4_. It is known that microbial production of CH_4_ (e.g. in soils, wetlands, lakes and manure slurries) responds positively to temperature [16,41]. However, the amount of CH_4_ emitted to the atmosphere depends also on the extent of microbial oxidation of CH_4_, which in soils depends on the oxygen availability, soil moisture, as well as on temperature [42]. The dependence of CH_4_ emission on soil moisture is nonlinear; if soils are too wet gas diffusion can be impeded; however, too dry soils can mean that the oxygen availability is generally high and thus CH_4_ is oxidized before reaching the atmosphere [43].

One type of microbial emission that is sensitive to temperature, and is abundant in the Netherlands, is that from manure, accounting for 0.15 Tg y^−1^ (14%) of the total national source and is the fourth most important source after enteric fermentation, fossil fuels and lakes (based on our prior estimates, see electronic supplementary material, figure S10). The temperature dependence of CH_4_ emission from manure slurries is comparable to that of wetlands with a Q10 factor of 3.4 [16]. Based on the observed temperature anomaly for April of 2°C above the mean of 9.4°C, and using the algorithm for calculating manure emissions of Sommer *et al*. [44], we estimate that manure emissions may have increased by a factor of 1.4 (see electronic supplementary material, information for the full calculation). Given that the mean manure source is 0.15 Tg y^−1^, the increase in April could be 0.06 Tg y^−1^ or 40% of the observed CH_4_ anomaly. Another contributing factor to the anomaly could be mineral soils. The land surface model, LPX-Bern, produces significant CH_4_ emissions from wet and inundated mineral soils throughout the year in the Netherlands (annual mean 0.08 Tg y^−1^), whereas our prior estimate, using the model JSBACH-HIMMELI, does not. One important difference that may explain this difference is that inundated area is determined dynamically in LPX-Bern, whereas it is fixed in JSBACH-HIMMELI (based on that used in the GCP CH_4_ budget [32]). In April, LPX-Bern also finds a small positive anomaly in the soil emissions, of 0.015 Tg y^−1^, which could explain 10% of the total anomaly. Although there is still considerable uncertainty in the LPX-Bern estimates, the anomaly is consistent with the inversion results. Lastly, we consider the effect of lakes using a process-based model, which combines a Mechanistic-Stochastic Model [45] for regional C-N-P dynamics [40] with a CH_4_ module relying on the Canadian Small Lake Model (CSLM) for the lake physics [46]. With a 2°C increase in April, the model predicted only a 3% increase in CH_4_ emissions for that month because it takes time for heat to propagate from the surface to depth and because April is still early in the season, i.e. before there is strong productivity and oxygen levels start to deplete. However, if a 2°C anomaly is maintained over the summer, CH_4_ emissions increase by a total of 18%, suggesting that there is potential for increased emissions from lakes with warmer summers.

It is noteworthy that although similar temperature anomalies occurred in other parts of Western and Central Europe in spring, the emission anomaly was localized in the Netherlands. This may be simply due to the fact that the total flux per unit area in the Netherlands is much higher (4–5 times) compared to other parts of Western and Central Europe (electronic supplementary material, figure S10) and, therefore, any anomaly may have been too small to be detected by the inversions with the current observation network. In addition, the emissions from mineral soils (according to the LPX-Bern estimates) and lakes (according to our prior estimate) are significantly higher in the Netherlands compared to the rest of Western and Central Europe (electronic supplementary material, figure S11).

In May to August, we found no significant CH_4_ anomaly for the Netherlands despite positive temperature anomalies. This may be because during this period, there was a strongly negative soil moisture anomaly, which may have led to enhanced microbial CH_4_ oxidation in soils, compensating any enhancement in CH_4_ production. In landfills, it has been found that CH_4_ emissions are negatively correlated with temperature in dry soil conditions [17,47]. Enhanced soil oxidation may also be the cause of the negative CH_4_ anomaly in September to October, when there was a strong soil water deficit but little to no temperature anomaly. The LPX-Bern model also shows a negative anomaly for soil emissions of 0.04 Tg y^−1^ equivalent to 13% of the October anomaly. The March CH_4_ anomaly, however, is somewhat of a puzzle as the temperature was slightly cooler than usual, and there was no soil moisture anomaly; therefore, we speculate that this anomaly may have been due to an abiogenic source. Moreover, we cannot exclude the contribution of abiogenic sources e.g. fugitive emissions from fossil fuels, to the positive anomaly in April.

The Mediterranean (except the Iberian Penninsula) also experienced a warmer than average spring. However, in contrast with north of the alps, the summer was wetter than usual, particularly in the north of the Iberian Penninsula and in the south and west of the Balkans, but with no temperature anomaly (figure 1). Falling in these regions with a wetter summer is Serbia, where we found a positive CH_4_ anomaly for much of the spring, summer and autumn. The anomaly for April–May coincided with elevated temperature (with no soil moisture anomaly), while that for July coincided with elevated soil moisture (but no temperature anomaly). In October, the positive CH_4_ anomaly may be explained again by a positive temperature anomaly, although the soil moisture was lower than usual for this month, but not lower than typical for the summer months. The reason for the positive anomaly in November–December, however, is unclear. LPX-Bern shows significant CH_4_ emissions in northern Serbia (where the anomaly detected by the inversions was most predominant) from wet mineral soils most of the year, and a positive anomaly in summer 2018 of 0.03 Tg y^−1^ or 25% of the July anomaly (electronic supplementary material, figure S11). In contrast with our inversion results, however, LPX-Bern indicates a negative anomaly for autumn 2018, which is probably driven by the lower soil moisture.

Although Fennoscandia was also affected by the heatwave and drought, we found no CH_4_ anomaly for this region. Within Europe, northern Fennoscandia is the only region with significant emissions from peatlands. Even though peatland emissions are known to be sensitive to temperature and soil moisture [48], the reason why no CH_4_ anomaly was detected could be twofold: (i) the largest temperature anomalies were in southern Fennoscandia, whereas northern Fennoscandia, where most of the peatlands are located, experienced temperatures that were less than two standard deviations warmer than the mean. Furthermore, there is evidence that there may have been contrasting effects of water table depth and temperature on CH_4_ emissions across peatland sites [49]. Rinne *et al*. [49] examined measurements at four peatland sites and found a reduction in the water table depth and thus CH_4_ emissions in 2018 at some sites (but not all) owing to differences in local topography, meaning that the area integrated change in emissions may have been small. (ii) Northern Fennoscandia is not well covered by the observation network and any signal may have been too small to be detected.

Despite the extreme meteorological conditions in 2018, we do not find any CH_4_ anomaly at the European scale, and Serbia was the only region where we detected an anomaly in the annual mean source. On the other hand, our results show that CH_4_ emissions are sensitive to changes in temperature and soil moisture, even in regions, such as the Netherlands, where natural emissions from e.g. wetlands are relatively small. In general, we found evidence that an increase in soil moisture, with little to no change in temperature, can lead to an increase in CH_4_ emissions, as was the case for Serbia. By contrast, warmer and drier conditions, as experienced by most of Europe north of the Alps in summer 2018, will likely not lead to any significant changes in CH_4_ emissions as the effects compensate each other. Summers in Europe are predicted to become hotter and drier in the future [50]; however, based on our results for 2018, we do not expect this to have a large effect on CH_4_ emissions.

It is also interesting to note that despite the considerable uncertainties in emission estimates from atmospheric inversions, the anomalies in 2018 were captured reasonably consistently by at least three inversions in our study. The uncertainties in inversion estimates could be further reduced by better-constrained boundary conditions in regional inversions, reduced atmospheric transport errors and a better-constrained atmospheric sink, as has also been shown in previous studies [37,51].

## 5. Conclusion

Despite the extreme meteorological conditions in 2018, we find that the CH_4_ emissions for most of Europe were similar to mean levels for 2005–2017. However, we do find some short-lived impacts on CH_4_ emissions at national scale. Specifically, we find a small positive anomaly in the emissions in April, which coincided with positive temperature and soil moisture anomalies, and a negative emission anomaly in September–October, which coincided with a negative anomaly in soil moisture. By contrast, we did not find any anomaly for the months May to August, most likely because the elevated temperature and low soil moisture had compensating effects on the CH_4_ emissions from biogenic sources, and we found no change in the annual emission with respect to the mean for 2005 to 2017. Although other regions of Western and Central Europe experienced similar meteorological extremes, changes in CH_4_ emissions were not apparent possibly owing to the fact that the per area fluxes, and, in particular, the mineral soil and lake emissions, were generally much lower than in the Netherlands. We also found significant anomalies for Serbia, with positive CH_4_ emission anomalies for the spring, summer and late autumn. Serbia experienced a warmer spring than usual, but in contrast with north of the Alps, the summer was wetter than usual. The anomaly for April–May coincided with elevated temperature, while that for July coincided with elevated soil moisture. The changes in emissions may be from mineral soils. Moreover, the annual emission for Serbia for 2018 was higher by 0.13 ± 0.15 Tg y^−1^ (33 ± 38%) than the mean for 2005 to 2017.

These results indicate that there is some sensitivity of CH_4_ emissions in Europe to meteorological conditions, which may in extreme cases even affect the annual national emissions, as was the case for Serbia in 2018. In such cases, it may be important to consider the effects of meteorology on CH_4_ emissions when comparing top-down estimates with emission inventories to avoid erroneously attributing changes in emissions due to meteorology to changes in human activity. Overall though, at the European scale, the variability of the emissions from 2005 to 2018 was small, and there was negligible impact on the annual emissions from the heatwave and drought experienced in 2018 north of the alps.

## Data accessibility

Atmospheric observations used in the inversions are available from the following databases: INGOS and ICOS data: https://www.icos-cp.eu, WDCGG data: https://gaw.kishou.go.jp, NOAA ESRL data: https://www.esrl.noaa.gov/gmd/, and EBAS data: http://ebas.nilu.no. Personal communications of data not included or only partially include in these databases (specifically data from IC3, LSCE, HMS, AGH and Univ. of East Anglia) are available from the station principal investigators listed in the Acknowledgements. The gridded CH_4_ fluxes and total emissions for each country from the inversions are available on Zenodo: https://doi.org/10.5281/zenodo.4719233. The LPX-Bern data are available upon request from Jurek Müller, Climate and Environmental Physics, University of Bern (jurek.mueller@climate.unibe.ch). The lake flux data are available upon request from Pierre Regnier, Université Libre de Bruxelles (Pierre.Regnier@ulb.ac.be). The JSBACH data are available upon request from Tuula Aalto, Finnish Meteorological Institute (Tuula.Aalto@fmi.fi). Access to flux data provided by the GCP is described in the references provided in the main text.
